# Proteasome and Organs Ischemia-Reperfusion Injury

**DOI:** 10.3390/ijms19010106

**Published:** 2017-12-30

**Authors:** Joan Oliva

**Affiliations:** Department of Medicine, LA BioMed at Harbor UCLA Medical Center, Torrance, CA 90502, USA; joliva@labiomed.org; Tel.: +1-424-571-7639

**Keywords:** ischemia-reperfusion injury, proteasome, organs, inflammation

## Abstract

The treatment of organ failure on patients requires the transplantation of functional organs, from donors. Over time, the methodology of transplantation was improved by the development of organ preservation solutions. The storage of organs in preservation solutions is followed by the ischemia of the organ, resulting in a shortage of oxygen and nutrients, which damage the tissues. When the organ is ready for the transplantation, the reperfusion of the organ induces an increase of the oxidative stress, endoplasmic reticulum stress, and inflammation which causes tissue damage, resulting in a decrease of the transplantation success. However, the addition of proteasome inhibitor in the preservation solution alleviated the injuries due to the ischemia-reperfusion process. The proteasome is a protein structure involved in the regulation the inflammation and the clearance of damaged proteins. The goal of this review is to summarize the role of the proteasome and pharmacological compounds that regulate the proteasome in protecting the organs from the ischemia-reperfusion injury.

## 1. Introduction

Since the first organ transplant performed by Joseph Murray and David Hume in 1954, organ transplantation techniques have been further developing all over the world. Successful Transplantation of vital and commonly demanded organs, such as livers, kidneys and hearts, is the main goal when addressing the issue of organ failure. The gap between the number of organ donors and the patients’ waiting list for organ transplantation is increasing. In the USA, this gap was around 60,000 patients in 2003, and in 2015, this gap increased to around 80,000 (Source: US Department of Health & Human Services). Because of the chronic shortage of organ donors, the transplantation of cadaveric organs became a major concern. To increase the rate and efficacy of transplantation, fresh organs should be transplanted to the patient; however it is not always possible to transplant freshly recovered organs. One of the most important steps in organ transplantation was the development of solutions of preservation solution to protect the organ until the transplantation.

Every year, the American Journal of Transplantation provides updated data on the transplantation of major organs such as livers, hearts, lungs, pancreases, kidneys, and intestines. From 100 patients, 69% received a liver from deceased donors, 6.9% from living donors, while the remaining percentage still remains on the waiting list, or has been removed due to death [1].The percentage of deceases donors is around 2.85% for the heart [2], 64% for the pancreas [3], 69.7% for the kidney [4], and 72% for the lungs [5]. These data show the importance of preserving organs from deceased donors, who compose the largest group of donors in the USA. The word ischemia comes from the Greek *iskhein* meaning restrict, and *emia* from the Greek word meaning blood. Ischemia is the deficiency of blood supplies in organs, due to blood vessel obstruction, or in the case of organs transplantation, the absence of blood vessels supplying the organ. The preservation of organs involves different steps: ischemia for the preservation period and reperfusion to reactivate the organs before transplantation. Reperfusion of the organs is the reestablishment of the blood flow and the reoxygenation of the organs. The ischemia-reperfusion steps damaged the organs by different factors such as decrease of ATP levels, decrease of nutrients, inflammation, and oxidative stress. The ischemia-reperfusion injury is a major problem for the outcome of a long term graft. After the transplantation, if the inflammatory reaction persists over the time, an interstitial fibrosis can develop which can have a negative impact on the graft outcome [6]. Two different approaches were developed for organ preservation: warm and cold ischemia-reperfusion (IRI). Warm IRI is used usually to preserve for a short time (30–60 min) before the organ transplantation. During warm IRI, the hepatic blood supply is interrupted [7], damaging hepatic cells, followed by hepatocytes and sinusoidal endothelial cell death. An extended warm IRI can partially or completely damage the organ, having a negative impact on the transplantation outcome, such as it was demonstrated with heart ischemia-reperfusion [8]. Because of the damage caused by the IRI, different preservation solutions and compounds were developed to attenuate the injuries, during the IRI: Institut Georges Lopez-1 (IGL-1), University of Wisconsin (UW), Histidine-tryptophan-ketoglutarate (Custodiol HTK), Belzer’s MPS [9,10]. Many works have been done and published about the composition of the preservation solution, but very few publications mention the relationship between the proteasome and the organ reperfusion-ischemia injury. The proteasome is a multiprotein complex regulating many cellular functions such as protein degradation, cell cycle, immune response, etc. The eukaryotic 26S proteasome is formed by three large multiprotein complexes: two 19S regulatory complex and one 20S core complex [11]. The activity of the 26S proteasome can be ATP independent and ATP dependent. During ischemia, the cellular ATP levels decrease, leading to the decrease of the 26S proteasome activity. In 2000, Buchan et al. noticed that the addition of the proteasome inhibitor CVT-634 alleviated the size of the infarct in a rat cerebral ischemia [12]. The focus of this review will be the role of the proteasome during IRI and the use of pharmacological compounds to modulate the activity of the proteasome such as MG-132, bortezomid, lactacystin, and epoxomicin.

## 2. Proteasome Generalities

The proteasome was discovered in 1978, by Dr. Rose, Dr. Hershko, and Dr. Ciechanover, who were rewarded with the Nobel Prize in Chemistry in 2004 [13]. The proteasome is a common complex for all living cells, necessary to recycle and eliminate unwanted proteins [14]. The proteasome pathway is involved in many cellular levels such as protein degradation, antigen processing, cell cycle, apoptosis, DNA repair and transcription, differentiation, immune response, etc. [15,16,17]. The 26S proteasome is present in the cytoplasm of every cell and also in the nucleus. It is usually formed by one 20S proteasome complex and two 19S proteasome complexes. However, different forms of the proteasome can be detected in the cells: 26S, 30S, immunoproteasome, and hybrid.

The 20S and 19S proteasome complex are composed of proteases and structural units. The assembly of alpha, beta, and ATP-dependent proteases is an ATP dependent mechanism, as there is degradation of the polyubiquitinated-proteins by the 26S proteasome. Proteins fated to be degraded by the 26S proteasome are usually poly-ubiquitinated on a lysine residue, before being targeted by the 26S proteasome [18]. All the steps required for the degradation of poly-ubiquitinated proteins are ATP-dependent, because their polyubiquitination and depolyubiquitination requires ATP [19,20]. In case of ATP depletion or low level in the cells, the 26S proteasome formation and the ubiquitin-dependent protein degradation are impaired, slowed down, or just absent. However, the ubiquitin-independent pathway activity will be increased, especially during the oxidative stress period, to compensate the decrease of the ubiquitin-dependent pathway [21]. The non-degradation of protein will lead to their accumulation in the cytoplasm, forming protein aggregations such as Mallory-Denk Bodies, and Lewy bodies [22,23]. A Similar phenotype can be observed during aging, when the activity of the proteasome decreases, and the accumulation of proteins can be observed, in different types of tissues such as the brain (Alzheimer’s, Parkinson’s) [24,25]. The accumulation of proteins in the cytoplasm becomes toxic for the cell, leading to pathology development and eventually results in death.

Polyubiquitination of the proteins require specific proteins involved in the conjugation of the ubiquitin to the targeted protein: E1 (ubiquitin-activating), E2 (ubiquitin-conjugating), and E3 (ubiquitin-ligating) enzymes [26,27]. It is important to notice that all mechanisms surrounding the 26S proteasome is regulated by the ATP which are low during the ischemia period, such as assembly of the proteasome and degradation of ubiquitinated proteins [28]. After the proteolysis, the ubiquitin proteins are released out of the 26S proteasome to be recycled for protein ubiquitination. All mechanisms surrounding the 26S proteasome (assembly, protein degradation, and activity) are regulated by the ATP, and by post-translation modification of the proteasome subunits [28]. The subunits of the proteasome can also post translationalated modified (phosphorylation, acetylation, myristoylation) [29,30,31].

Oxidative stress is an imbalance between the levels of anti-oxidant and pro-oxidant species. In normal conditions, reactive oxygen species (ROS) are produced as the result of the oxidative metabolism of the cells. ROS are capable to oxidize proteins, lipids, and nucleic acids, which can explain that ROS is associated with aging, diseases development (cancer, neurodegenerative diseases), and cellular dysfunctions [32]. Because the production of ROS is inevitable during the aerobic metabolism, two major mechanisms were developed by the cells as an antioxidant defense system to protect the cell: (1) the production of proteins having an anti-oxidant functions; and (2) the increase of the production of the 20S proteasomes to degrade oxidized proteins. When ROS levels are increasing, a key protein nuclear factor erythroid 2 (Nrf2) is activated and increases the production of antioxidant proteins but also the production of the proteasome subunits [33]. The levels of ROS are counterbalanced by anti-oxidant molecules such as superoxide dismutase (SOD), glutathione S-transferase (GST), catalase, and peroxidase [34]. In parallel, the formation of 20S proteasome is increased to degrade oxidized proteins [35]. However, when the levels of ROS are superior to the anti-oxidant molecules level, the cells undergo an oxidative stress and oxidized proteins aggregate into a toxic protein complex resistant to proteolysis called aggresomes [32]. These aggresomes can be detected in neurodegenerative diseases or liver diseases [36,37]. During the reperfusion step, the production of ROS and the formation of oxidized proteins is increased because of the sudden oxygen supply, indicating that proteasome activation during the reperfusion step could reduce the toxic accumulation of oxidized proteins.

## 3. Proteasome and the Oxidative Stress

Over the past millions of years, eukaryotic cells developed a highly efficient mechanism to produce energy: aerobic metabolism [38,39]. The aerobic metabolism is necessary for the production of ATP, but in return, the aerobic metabolism produces reactive oxygen species, which are toxic for the cell. The organisms are exposed to ROS their whole life, which lead to the development of antioxidant defenses that were mentioned in part 1.

The 26S proteasome is part of this antioxidant cellular defense. Peroxisomes and endoplasmic reticulum are also sources of ROS, which can be toxic if their cellular levels are high [40,41]. At physiological levels, ROS is not felt as a stress by the cell and they are cleared by antioxidant proteins, such as superoxide dismutase, catalase, glutathione peroxidase, glutathione transferase, thioredoxin, and peroxiredoxin [34]. In 1973, Hearse et al. suggested that the reoxygenation of the organs, after the ischemia, was probably a source of injury for the organ [42]. All organs have in common injuries characteristic of such an increase of the number of oxidized proteins, inflammation, or cell death. In a langendorff isolated rat heart, heart ischemia resulted in the decrease of the enzyme SOD and glutathione peroxidase. However, it was surprising that the expression of SOD and glutathione peroxidase continued to decrease after the re-oxygenation of the heart, when it was expected that the oxygen should reverse the ischemia effect on their expression [43]. The re-oxygenation of the heart increased the lipid peroxidation levels, decreased the levels of glutathione and thiols groups, compare to the control. The reperfusion of lungs also increased the oxidative stress, compared to the lungs before the reperfusion [44], which was reduced by the use of *N*-Acetyl-Cysteine, an antioxidant enzyme [45]. The re-oxygenation of hearts leads to the oxidation of protein tyrosine phosphatases (PTP), involved in the dephosphorylating of receptor tyrosine kinase (RTK) [46]. The oxidation of PTP results in a loss of function and the development of diseases such as Leopard syndrome, severe combined immunodeficiency [47,48]. The inactivation of PTP is associated with the activation of RTK which plays a role in the injury after reperfusion. Endothelin 1 receptor is activated when PTP is inactive [49], and it was shown that endothelin 1 plays a role in the heart injury by inducing heart fibrosis and in the lungs, after the reperfusion [50]. Other mechanisms connecting the ROS and IRI were published. For example, the ROS increase in liver induced cell death by activating the tumor necrosis factor receptor [51] or by increasing the inflammatory response by activating IL-4 [52]. Using large scale gene or protein screening, some publications show a difference of gene expression of oxidized protein pattern between ischemic tissue and ischemic-reperfused tissue. In general, less expressed or oxidized proteins localized in the mitochondria were detected NADH dehydrogenase, succinate dehydrogenase, voltage-dependent anion channel [53,54]. In a lung IRI male model, Ikejiri showed that 79.7% of genes related with the oxidative stress were upregulate and 20.2% of them were downregulated [55]. These genes are related with the production of ROS or of antioxidant enzyme, with no other precision about their identity [55].

The uncleared ROS compounds can still oxidize proteins, which can be rescued by specific repair pathways developed by the cells [56,57]. When the ROS levels slightly increase in the cells, the activity of the 26S proteasome increases to remove oxidized proteins, using an ubiquitin-dependent degradation pathway [58]. However, when the level of the oxidative stress is high, the 26S proteasome is dismantled, and oxidized proteins level rise but these proteins will be degraded by the 20S proteasome. The degradation of oxidized proteins is ATP and ubiquitin independent [21,59]. Oxidized proteins are unfolded and targeted to the 20S proteasome complex, allowing the cell to clear oxidized, which can be toxic for the cell [60]. However, when the oxidative stress becomes chronic, the number of oxidized proteins becomes too high to be cleared by the proteasome and they accumulate in aggresomes. Also, chronic oxidative stress comes with an inflammation. During the inflammation response, cytokines such as Interferon gamma (IFNγ) and Tumor Necrosis Factor alpha (TNFα) are released. These cytokines induce the production of the specific subunits of the immunoproteasome (LMP2, LMP7, and MECL-1). The consequence is the switch of the 26S proteasome population to the formation of immunoproteasome [61]. It was reported that the IκBα, a protein blocking the activity of nuclear factor kappa-light-chain-enhancer of activated B cells (NFκB), can be degraded by the 20S proteasome during the cellular oxidative stress [62]. The degradation of IκBα releases NFκB which can migrate into the nucleus to activate the anti-inflammatory response [63]. NFκB will target the promoter of pro-inflammatory cytokines and induces their synthesis, such as TNF-α, IL6, COX2 [64]. Cytokines IFNγ and TNFα increase the synthesis of LMP2, LMP7, and MECL-1 (specific subunits of the immunoproteasome) which replace the proteasome subunits B1, B2, and B5 [65]. LMP2, The major role of the immunoproteasome is to process antigens for the major histocompatibility complex (MHC) class I on T lymphocytes [66]. The switch of 26S proteasome to the immunoproteasome formation leads to the decrease of 26S proteasome activity [67].

Two mechanisms can explain the accumulation of the oxidized proteins in the cells: decrease of the proteasome activity during aging and/or the saturation of 26S proteasome to clear oxidized proteins [68,69]. The formation of aggresomes can be observed in aging related diseases such as Alzheimer’s, Parkinson’s, and Huntington disease [70,71,72], due to the decrease of the 26S proteasome activity, leading to an accumulation of oxidized proteins [73]. However, it is not known if oxidative stress, inflammation, or proteasome are involved in the disease development. The most realistic global mechanism is that all factors might be involved, and their effect is cumulative, but the appearance of the symptoms takes a very long time to appear. In the case of Alzheimer’s disease, the decrease of the 26S proteasome activity is associated with the accumulation of the proteins βamyloid and Tau, but also with the formation of aggresomes which are toxic for the cells if they are not cleared. Similar mechanisms were found in the liver, with the formation of Mallory-Denk Bodies (MDBs). Oliva et al. published an in vitro study with Hepg2 cells that, under a chronic inflammation treatment, aggresomes formed in the cells [74]. Dr. French showed the relationship or similarity between Alzheimer aggresomes and MDBs [75]. The causes of the oxidative stress arise from different origins such as stress, alcohol intake, sick liver, infections [76,77,78].

One therapeutic approach to reverse or delay aging related diseases will be to activate the proteasome to degrade the aggresomes and oxidized proteins. Unfortunately, no activator of the proteasome has been discovered so far. It is important to notice that some publications report the activation of the 26S proteasome by using inhibitors of the proteasome such as, bortezomid [79].

## 4. Proteasome and Organs Ischemia-Reperfusion Injury

To our knowledge, only 176 publications are found when the words “proteasome ischemia reperfusion” are used to research publications related with the proteasome and the ischemia reperfusion, in Pubmed, in November 2017. The actual data about the potential beneficial role of the proteasome inhibitors (PI) and the positive outcome of the organ transplantation are very limited. However, the initial results seem promising considering the use of PI. All the organs during the IRI share the same type of injuries: inflammation; impairment of microvascular function; and cell death (apoptotic and necrotic).

If the inflammatory response is chronic, it can lead to the development of fibrotic tissue, which impairs the long-term graft success. For example, the reperfusion of the kidneys increases the recruitment of neutrophils into the kidney, which will exacerbate the inflammatory reaction [80]. Yago et al. [80] show that blocking neutrophil integrins prevent the injuries due to the reperfusion, which are mediated by the key factors such as ROS and activated NFκB. NFκB is a key factor in inflammatory reaction activation, and the amplification of the inflammatory reaction [81]. The activation of NFκB requires the ubiquitination and the degradation of the protein IκB [82]. Blocking the degradation of the IκB by using PI prevents the activation of the NFκB pathway involved in the production of cytokines such as IL-6 and TNFα [83]. It was observed in cold liver preserved, TNFα was released, contributing to the inflammatory response [84], and the use of bortezomib in cold preserved liver decrease the presence of TNFα and IL1-β [85]. Produced and released TNFα can also activate NFκB, amplifying the inflammatory response with a positive back-loop [86]. The inflammatory response due to the reperfusion was also detected for the heart IRI [87,88]. PI offers additional tools that could prevent or at least decrease the inflammatory reaction due to the reperfusion, which can have a negative impact on the long term graft.

During the ischemia, the disruption of the blood flow causes the vasoconstriction of the blood vessel and the reperfusion injury is associated with the endothelial cell dysfunction. Vascular resistance is increased in organ ischemia, because eNOS is decreased. eNOS is well known to have a protective effect on the endothelial cells by inducing the vasodilation of the blood vessel. In a ischemia vasoconstriction liver cold ischemia reperfusion injury, Zaouali et al. showed that the addition of bortezomib increased the production of eNOS and decreased the vascular resistance, improving the blood flow through the liver [85]. The injury of the endothelial cells promotes the low perfusion and induces the graft vasculopathy, which is associated with a low long-term graft outcome [89].

**Scheme 1:**
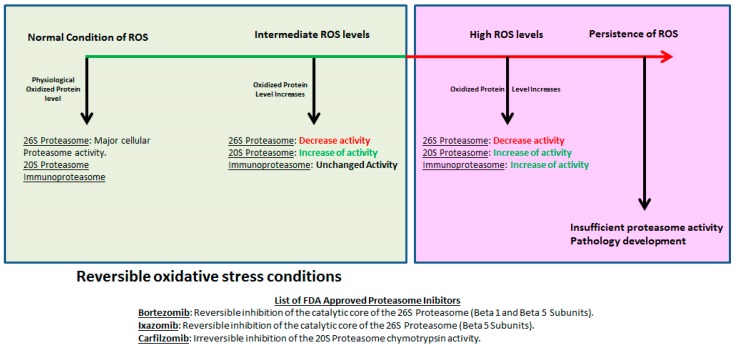
Relation between the reactive oxygen species (ROS) levels and the proteasome activities (20S, 26S and immunoproteasome). At the bottom of the scheme, list of the Food and Drug Administration (FDA) approved proteasome inhibitors and they mode of actions on the proteasomes.

The treatment of many organs with inhibitors of the proteasome was reported, and the beneficial effect on decreasing the negative effects of the organ reperfusion. The Scheme 1 shows the relationship between the ROS levels and the activities of the proteasome, and also the list of proteasomes inhibitors approved by the Food and Drug Administration (FDA). Only three proteasome inhibitors were approved by the FDA: Bortezomid, in 2003; Carfilzomib, in 2012, and Ixazomib, in 2015.

### 4.1. The Brain

The first publication mentioning the relationship between the proteasome activity and the IRI was published in 1996. The proteasome activity was recovered after reperfusion of the gerbil cortex [90]. However, it was not until 2000 that Buchan reported that the use of proteasome inhibitor can reduce the infarct volume in a rat cerebral ischemia, and that laboratories started to look at the relationship between the proteasome and the ischemia-injury [12]. This cerebral infarct was caused by the activation of NFκB which triggered a neuro-inflammatory response. NFκB was activated when IκB was degraded by the proteasome during the ischemia [91]. It was reported that during the ischemia-injury, NFκB translocated into the nucleus [92], which was identified to be a factor of brain injury after IRI [93]. NFκB in the nucleus increased the expression of pro-inflammatory, which was blocked by PS519 proteasome inhibitor. PS519 decreased the inflammatory response, the neutrophil and macrophage infiltration, but helped the brain recover more neuronal activity compared to the untreated rats [93]. The inhibition of NFκB reduced the expression of pro-inflammatory NFκB target genes such as ICAM-1 [94], E-Selectin [95], TNF-α [96], and IL-1β [97]. As mentioned above about proteasome generalities, the 26S proteasome population switches to the immunoproteasome in case of inflammatory reaction. This switch of proteasome population was observed during a short brain ischemia period [98]. The switch resulted in the decrease of the 26S and 20S proteasome activity, and an increase of the immunoproteasome activity [98]. In 2006, a reversible proteasome inhibitor was used to protect rat brains against cerebral ischemia. Velcade (also called PS-341 or Bortezomid) was injected immediately after cerebral ischemia was induced [99]. When Velcade was injected 1 and 2 h after cerebral ischemia (not at 3 h), the authors observed the positive impact of the Velcade treatment in decreasing the infarct volume, in two different rat models Wistar-Kyoto and Sprague-Dawley rats. As mentioned in the introduction, during the aging process, the activity of the proteasome decreases is accompanied by the accumulation of proteins in aggresomes which are toxic for the cells. Along with aging, the probabilities of having a stroke increases, which would result in the shortage of blood, oxygen, and nutrients to the brain. The use of Velcade was shown to have beneficial effects in preventing damages due to a stroke, in aging population. In 2010, L. Zhang showed that the treatment of stroke Wistar rat model reduced damaged brain areas by decreasing the infarct volume and helped to break down the blood clots in the brain blood vessels [100]. An excellent review written by Caldeira et al. resumes the importance of the proteasome and the regulation of the proteasome by pharmacological compounds in decreasing the negative effect of brain IRI [101]. In summary, the use of proteasome inhibitor decreases the infarct volume, the inflammatory response, and the neutrophil infiltration in the areas of the brain deprived of oxygen.

### 4.2. The Heart

In 1999, Campbell et al. used a proteasome inhibitor PS-519 to improve the recovery of rat hearts after ischemia reperfusion [102]. Compared to the control ischemia reperfusion rats, rats treated with PS-519 improved the coronary flow and the left ventricle pressure while preserving cardiac contractile function [102]. In addition, the presence of polymorphonuclear leukocytes (PMN) was reduced, which is a significant result because the tissue damage and the pathophysiology of the ischemia are increased by the PMN infiltration [103]. It was the first publication mentioning the potential role of pharmacological treatment of the heart during IRI, to decrease the negative side effects of the IRI [102]. Because it is well established that oxidative stress is a consequence of the ischemia, one of the key players of the ischemia-reperfusion damaging protein that researchers focused on was nuclear factor kappa-light-chain-enhancer of activated B cells (NFκB) [104]. During the oxidative stress, NFκB is translocated into the nucleus, in absence of the degradation of the IKb [104], and induces the production of pro-inflammatory cytokines (IL-6, IL-1, TNFα, COX2, IFNγ). Because of the major role of NFκB in tissue damage after reperfusion, Bao et al. decided to use proteasome inhibitors PR-39 and PR-11 to block the degradation of IκB [91]. They succeeded to block the degradation of IκB and to decrease the heart damage in rats, perfused for 24 h. Compare to the control, they noticed also a reduction of the infarct size, an improvement of the blood pressure, and the relaxation and contractibility of the heart. It was reported that the use of proteasome inhibitor decreases the neutrophil infiltration, reducing by consequence the inflammatory response during the reperfusion [93]. Similar results were reported by Pye et al. in 2003 [105]. In 2005, it was reported that the accumulation of ubiquitinated proteins after ischemia was abolished when the hearts were pretreated with the inhibitor of the proteasome MG-132. In general, the use of inhibitors of the proteasome of many categories such as PS-519, PR-11, MG-132 has a protective effect on the heart after the IRI.

### 4.3. The Liver

In the 60s, Dr. Brown and Dr. Mc Dermott developed a cold method for liver preservation [106]. This approach dramatically improved the survival rate of the patients. The survival rate of patients 1 year and 5 years after liver transplantation is 95% and 75%, respectively [107]. Once the liver is removed from the body, the oxygen and nutrient supplies are interrupted and the aerobic metabolism switches to anaerobic metabolism. The first consequence was the decrease of ATP levels, and the shutting down all ATP-dependent mechanisms such as ionic pumps, chromatin remodeling, Ca^2+^ storage, and protein degradation. Livers, destined for transplantation, were preserved in cold conditions and perfused with preservation solutions such as Institut Georges Lopez-1 (IGL-1), University of Wisconsin (UW), Histidine-tryptophan-ketoglutarate (Custodiol HTK), Belzer’s MPS (Machine Perfusion Solution). During prolonged ischemia when the liver is cold preserved in these solutions, the ATP levels decrease due to the absence of nutrients and oxygen. This decrease led to the disassembly of the 26S proteasome. The decrease of the 26S proteasome activity during the ischemia is reflected by the accumulation of unwanted proteins, which are toxic for the cell, if their accumulation is chronic [75]. Aggresome formation is a cell defensive mechanism to protect the cell from unwanted (damaged, unfolded) proteins which could migrate anywhere in the cells and impair cellular functions. Via microtubules, these toxic proteins gather in package forming aggresomes [108]. This mechanism is triggered during the cold liver ischemia but it will also happen during the reperfusion for two major reasons: the return of oxidative stress due to the blood flow restoration and the inflammatory reaction due to the oxidative stress.

The first mention of the potential relationship between the IRI in the liver and the proteasome was mentioned in 1996, in a turtle model [109]. The authors showed that the activity of the postglutamyl peptide hydrolytic-like was increased by around 30% after 20 h of ischemia and 24 h of aerobic recovery [109]. It was not until 2003 that the use of 3,4-dichloroisocoumarin (DCI), a serine-protease inhibitor to decrease liver damage after ischemia-reperfusion was reported [110]. The level of lactate dehydrogenase decreased by 21 times when DCI was added to the histidine-tryptophan-keroglutarate (HTK) preservation solution compared to HTK alone. It became evident that the proteasome plays a major role in the liver IRI, and the addition of proteasome regulating pharmacological compounds will similarly play an important role. As mentioned in this manuscript, the activity of the proteasome decreases with age. Because of the consequences of the decreased proteasome activity, scientists are working on the activation of the 26S proteasome, to delay aging [111], by developing molecules capable of activating it [112]. However, it was reported that inhibitors of the proteasome can also activate the 26S proteasome at a low-dose, when high doses are used to treat myeloma. Indeed, high doses of the bortezomib are usually used to block the activity of the proteasome, but by lowering the concentration, Bardag-Gorce et al. reported that the use of low non-toxic doses of bortezomid increased the activity of the 26S proteasome in a model of rat alcoholic liver disease. In parallel, while the 26S proteasome increased activity by the low dose of bortezomid, the authors noticed a decrease of the oxidative stress and an increase of antioxidant proteins [79].

Many proteasome inhibitors, reversible or irreversible, were developed to be more powerful and more specific in inhibiting the activity of the proteasome for myeloma treatment. The following list is not exhaustive but it gives a good idea of the number of proteasome inhibitors available on the market: PR-11, PR-39, MG-132, Carfilzomib, Delanzomib, Bortezomid, Ixazomib [113]. Yao et al. studied the effect of lactacystin on the liver, after a rat intestinal ischemia-reperfusion [114]. It is well established that during the reperfusion, the liver will be damaged and the damage level can be assessed. Different parameters should be measured such as the blood level of aspartate aminotransferase (AST), alanine aminotransferase (ALT), and serum albumin [115]. Lactacystin was proved to be efficient in decreasing liver injury after ischemia-reperfusion, and it was tested on a rat intestinal ischemia-reperfusion model. At 0.6 mg/kg, lactacystin decreased the level of AST and lactate dehydrogenase (LDH) back to the control level, and decreased the level of ALT by 23%. In addition, lactacystin decreased the level of ICAM-1 which is involved in the neutrophil infiltration, decreased the level of expression of NFκB which is involved in the pro-inflammatory response, and decreased the level of myeloperoxidase, an important enzyme that generates the high level of oxidant compounds in the cells [114]. This first study in liver ischemia-reperfusion showed the utility to test more proteasome inhibitors, to be used in treating IRI. MG-132 was also used in liver ischemia-reperfusion and showed a protective effect on the early phase of the reperfusion. MG-132 decreased the AST, ALT, and LDH levels, and altered the expression of antioxidant enzymes by decreasing the levels of the catalase and superoxide dismutase [116]. The activity of the 20S proteasome was also decreased by lactacystin, in the white blood cells [114], when the MG-132 decreased the activity of the 20S proteasome in the liver [116]. Another type of reversible proteasome inhibitor was used for steatotic liver ischemia-reperfusion: Bortezomid. At 0.1 mg/kg, a similar dose as for MG-132 and lactacystin, bortezomid reduced the activity of NFκB, the pro-inflammatory response (less TNFα and MIP-2 expressed) and the ALT levels [117]. At the same time, another laboratory published results leading to similar conclusions. The use of bortezomid decreased the activity of the proteasome in rat models of reduced-size for orthotopic liver transplantation [118]. The authors confirmed that bortezomid decreased the activity of the proteasome in the liver. In the presence of bortezomid at 0.1 mg/kg, the levels of ALT, AST, and glutamate dehydrogenase (GLDH) decreased as it was already mentioned by Alexandrova et al. [116,118]. Other liver injuries induced by the ischemia-reperfusion were reduced such as the lipid peroxidation, hepatocyte necrosis, neutrophil infiltration, inflammation, and the endoplasmic reticulum stress [118]. Matrix metalloproteinases (MMP) are proteins that control the extracellular-matrix of the cells, which is related with the inflammatory response and cell migration. MMP are also involved in liver injury after the ischemia-reperfusion [119], such as MMP-2 and MMP-9. The degradation of the extra cellular matrix by MMP-2 and MMP-9 was connected with the leukocytes infiltration, which also produce MMP-9, in a mice liver IRI model [120]. In another model, bortezomid at 0.1 mg/kg reduced the expression of MMP-2 and MMP-9 to basal levels, reducing also the leukocyte infiltration [121].

It was also noticed that the activity of the AMP-activated protein kinase (AMPK) is increased when the liver is placed in the preservation solution during ischemia. AMPK is a sensor of the level of ATP, whose activity increases when the cellular levels of ATP decreases [122]. It was previously reported that the activation of AMPK in heart and kidney was beneficial to protect the organs from IRI [123,124]. The activation of AMPK was also described in breast cancer, lung, and cervical cancer cell line, by treating the cells with bortezomid [125]. IGL-1 solution supplemented with bortezomid was used to preserved liver, from a rat ischemia-reperfusion model. The authors showed a higher activity of the AMPK by adding bortezomid, compared to the IGL-1 solution alone [126]. The increase of AMPK activity was correlated with a strong decrease of ALT, AST, GLDH, and malondialdehyde levels, indicating a decrease of liver injury after the reperfusion. The beneficial effect of AMPK might be due to the maintenance of a high level of cellular ATP [127]. Besides its role in the maintenance the energy homeostasis, AMPK is also involved in decreasing inflammation, apoptosis and oxidative stress. The activation of AMPK by the bortezomid in C2C12 murine myotubes decreased the inflammation and the ER stress [128]. The decrease of the inflammation is translated into the decrease of inflammatory cells infiltration, which are responsible of amplifying the inflammatory response.

## 5. Conclusions

Nowadays, three proteasome inhibitors have been approved by the Food Drug Administration for the treatment of myelomas: (1) Bortezomid was approved in 2003 to treat myeloma [129]; (2) Carfilzomib was approved in 2012 to treat refractory myeloma [130]; and (3) Ixazomib was approved in 2015 to treat also refractory myeloma [131]. The dose of bortezomid used to treat myeloma is around 509 ng/mL in the plasma (FDA sources). In the IRI animal models, the injected bortezomid dose is 440 times lower in average (0.1 mg/kg), compared to the 509 ng/mL in the patients plasma. The difference of dosage is reflected by the different activity and curative properties of the bortezomid.

Many common factors involved in organ injury can be blocked or reduced by the use of inhibitors of the proteasome, to decrease the IRI: the decrease of the 26S and 20S proteasome activity, the oxidative stress, the activity of NFκB, the inflammatory response and inflammatory cell infiltration. All these factors play a role in the liver IRI, but it is also important to mention a paper relating the proteasome activity during aging and liver IRI [132]. In an aging mice population, the activity of the proteasome decreased and the degradation of IκB also decreased during liver IRI, mainly because of the low expression of proteasome 26S subunit, non-ATPase 4 (PSMD4) [132]. PSMD4 is an important protein involved in the assembly of the 19S proteasome [132]. Aging is accompanied by the decrease of the proteasome activity, an increase of the oxidative stress and lipid oxidation, in the neuron [133,134,135]. A Similar injury pattern was observed between aging and the organs IRI, increasing the great potential of proteasome inhibitors to decrease aging and organs IRI. Because a large number of proteasome inhibitors are available on the market, the possibilities of simple or the combination of treatments could increase the probability of developing a solution to prevent IRI, and increase the outcome of transplantation.

The use of non-toxic low dose of proteasome inhibitors offers patients new hope and offers research laboratories new options for improving preservation solutions not only for the liver, and for others organs such as kidneys, and hearts, but also for potential use in neurodegenerative diseases related with aging.
